# The importance of human resources management in health care: a global context

**DOI:** 10.1186/1478-4491-4-20

**Published:** 2006-07-27

**Authors:** Stefane M Kabene, Carole Orchard, John M Howard, Mark A Soriano, Raymond Leduc

**Affiliations:** 1 Management and Organizational Studies, The University of Western Ontario, London, Ontario, Canada; 2 Schulich School of Medicine, The University of Western Ontario, London, Ontario, Canada; 3 School of Nursing, The University of Western Ontario, London, Ontario, Canada

## Abstract

**Background:**

This paper addresses the health care system from a global perspective and the importance of human resources management (HRM) in improving overall patient health outcomes and delivery of health care services.

**Methods:**

We explored the published literature and collected data through secondary sources.

**Results:**

Various key success factors emerge that clearly affect health care practices and human resources management. This paper will reveal how human resources management is essential to any health care system and how it can improve health care models. Challenges in the health care systems in Canada, the United States of America and various developing countries are examined, with suggestions for ways to overcome these problems through the proper implementation of human resources management practices. Comparing and contrasting selected countries allowed a deeper understanding of the practical and crucial role of human resources management in health care.

**Conclusion:**

Proper management of human resources is critical in providing a high quality of health care. A refocus on human resources management in health care and more research are needed to develop new policies. Effective human resources management strategies are greatly needed to achieve better outcomes from and access to health care around the world.

## Background

### Defining human resources in health care

**Figure 1:**
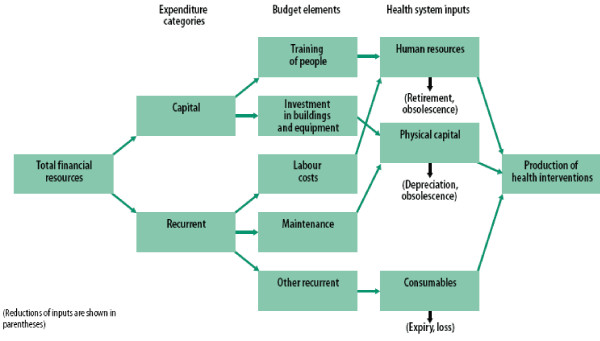
**Relationship between health system inputs, budget elements and expenditure categories**. Source: World Health Report 2000 Figure 4.1 pg.75.  Figure 1 identifies three principal health system inputs: human resources, physical capital and consumables. It also shows how the financial resources to purchase these inputs are of both a capital investment and a recurrent character. As in other industries, investment decisions in health are critical because they are generally irreversible: they commit large amounts of money to places and activities that are difficult, even impossible, to cancel, close or scale down [1].

Within many health care systems worldwide, increased attention is being focused on human resources management (HRM). Specifically, human resources are one of three principle health system inputs, with the other two major inputs being physical capital and consumables [1]. Figure 1 depicts the relationship between health system inputs, budget elements and expenditure categories.

Human resources, when pertaining to health care, can be defined as the different kinds of clinical and non-clinical staff responsible for public and individual health intervention [1]. As arguably the most important of the health system inputs, the performance and the benefits the system can deliver depend largely upon the knowledge, skills and motivation of those individuals responsible for delivering health services [1].

As well as the balance between the human and physical resources, it is also essential to maintain an appropriate mix between the different types of health promoters and caregivers to ensure the system's success [1]. Due to their obvious and important differences, it is imperative that human capital is handled and managed very differently from physical capital [1]. The relationship between human resources and health care is very complex, and it merits further examination and study.

Both the number and cost of health care consumables (drugs, prostheses and disposable equipment) are rising astronomically, which in turn can drastically increase the costs of health care. In publicly-funded systems, expenditures in this area can affect the ability to hire and sustain effective practitioners. In both government-funded and employer-paid systems, HRM practices must be developed in order to find the appropriate balance of workforce supply and the ability of those practitioners to practise effectively and efficiently. A practitioner without adequate tools is as inefficient as having the tools without the practitioner.

### Key questions and issues pertaining to human resources in health care

When examining health care systems in a global context, many general human resources issues and questions arise. Some of the issues of greatest relevance that will be discussed in further detail include the size, composition and distribution of the health care workforce, workforce training issues, the migration of health workers, the level of economic development in a particular country and sociodemographic, geographical and cultural factors.

The variation of size, distribution and composition within a county's health care workforce is of great concern. For example, the number of health workers available in a country is a key indicator of that country's capacity to provide delivery and interventions [2]. Factors to consider when determining the demand for health services in a particular country include cultural characteristics, sociodemographic characteristics and economic factors [3].

Workforce training is another important issue. It is essential that human resources personnel consider the composition of the health workforce in terms of both skill categories and training levels [2]. New options for the education and in-service training of health care workers are required to ensure that the workforce is aware of and prepared to meet a particular country's present and future needs [2]. A properly trained and competent workforce is essential to any successful health care system.

The migration of health care workers is an issue that arises when examining global health care systems. Research suggests that the movement of health care professionals closely follows the migration pattern of all professionals in that the internal movement of the workforce to urban areas is common to all countries [2]. Workforce mobility can create additional imbalances that require better workforce planning, attention to issues of pay and other rewards and improved overall management of the workforce [2]. In addition to salary incentives, developing countries use other strategies such as housing, infrastructure and opportunities for job rotation to recruit and retain health professionals [2], since many health workers in developing countries are underpaid, poorly motivated and very dissatisfied [3]. The migration of health workers is an important human resources issue that must be carefully measured and monitored.

Another issue that arises when examining global health care systems is a country's level of economic development. There is evidence of a significant positive correlation between the level of economic development in a country and its number of human resources for health [3]. Countries with higher gross domestic product (GDP) per capita spend more on health care than countries with lower GDP and they tend to have larger health workforces [3]. This is an important factor to consider when examining and attempting to implement solutions to problems in health care systems in developing countries.

Socio-demographic elements such as age distribution of the population also play a key role in a country's health care system. An ageing population leads to an increase in demand for health services and health personnel [3]. An ageing population within the health care system itself also has important implications: additional training of younger workers will be required to fill the positions of the large number of health care workers that will be retiring.

It is also essential that cultural and geographical factors be considered when examining global health care systems. Geographical factors such as climate or topography influence the ability to deliver health services; the cultural and political values of a particular nation can also affect the demand and supply of human resources for health [3]. The above are just some of the many issues that must be addressed when examining global health care and human resources that merit further consideration and study.

### The impact of human resources on health sector reform

When examining global health care systems, it is both useful and important to explore the impact of human resources on health sector reform. While the specific health care reform process varies by country, some trends can be identified. Three of the main trends include efficiency, equity and quality objectives [3].

Various human resources initiatives have been employed in an attempt to increase efficiency. Outsourcing of services has been used to convert fixed labor expenditures into variable costs as a means of improving efficiency. Contracting-out, performance contracts and internal contracting are also examples of measures employed [3].

Many human resources initiatives for health sector reform also include attempts to increase equity or fairness. Strategies aimed at promoting equity in relation to needs require more systematic planning of health services [3]. Some of these strategies include the introduction of financial protection mechanisms, the targeting of specific needs and groups, and re-deployment services [3]. One of the goals of human resource professionals must be to use these and other measures to increase equity in their countries.

Human resources in health sector reform also seek to improve the quality of services and patients' satisfaction. Health care quality is generally defined in two ways: technical quality and sociocultural quality. Technical quality refers to the impact that the health services available can have on the health conditions of a population [3]. Sociocultural quality measures the degree of acceptability of services and the ability to satisfy patients' expectations [3].

Human resource professionals face many obstacles in their attempt to deliver high-quality health care to citizens. Some of these constraints include budgets, lack of congruence between different stakeholders' values, absenteeism rates, high rates of turnover and low morale of health personnel [3].

Better use of the spectrum of health care providers and better coordination of patient services through interdisciplinary teamwork have been recommended as part of health sector reform [4]. Since all health care is ultimately delivered by people, effective human resources management will play a vital role in the success of health sector reform.

## Methods

In order to have a more global context, we examined the health care systems of Canada, the United States of America, Germany and various developing countries. The data collection was achieved through secondary sources such as the Canadian Health Coalition, the National Coalition on Health Care and the World Health Organization Regional Office for Europe. We were able to examine the main human resources issues and questions, along with the analysis of the impact of human resources on the health care system, as well as the identification of the trends in health sector reform. These trends include efficiency, equity and quality objectives.

## Results

### Health care systems

#### Canada

**Figure 2:**
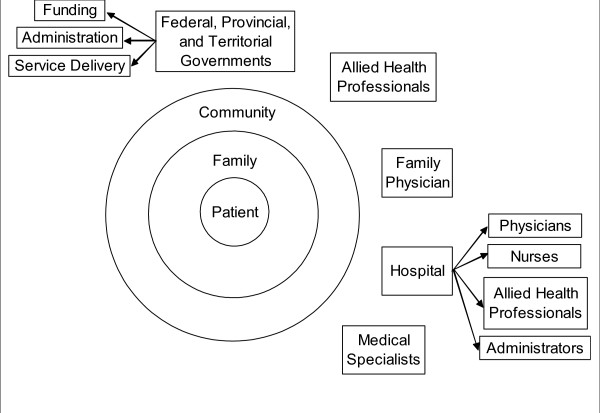
**Overview of the major stakeholders in the Canadian health care system**. Figure 2 depicts the major stakeholders in the Canadian health care system and how they relate.

The Canadian health care system is publicly funded and consists of five general groups: the provincial and territorial governments, the federal government, physicians, nurses and allied health care professionals. The roles of these groups differ in numerous aspects. See Figure 2 for an overview of the major stakeholders in the Canadian health care system.

Provincial and territorial governments are responsible for managing and delivering health services, including some aspects of prescription care, as well as planning, financing, and evaluating hospital care provision and health care services [5]. For example, British Columbia has shown its commitment to its health care program by implementing an increase in funding of CAD 6.7 million in September 2003, in order to strengthen recruitment, retention and education of nurses province-wide [6]. In May 2003, it was also announced that 30 new seats would be funded to prepared nurse practitioners at the University of British Columbia and at the University of Victoria [6]. Recently the Ontario Ministry of Health and Long Term Care announced funding for additional nurse practitioner positions within communities. Furthermore, most provinces and territories in Canada have moved the academic entry requirement for registered nurses to the baccalaureate level, while increasing the length of programmes for Licensed Practice Nurses to meet the increasing complexity of patient-care needs. Several provinces and territories have also increased seats in medical schools aimed towards those students wishing to become family physicians [7].

The federal government has other responsibilities, including setting national health care standards and ensuring that standards are enforced by legislative acts such as the Canada Health Act (CHA) [5]. Constitutionally the provinces are responsible for the delivery of health care under the British North America (BNA) Act; the provinces and territories must abide by these standards if they wish to receive federal funding for their health care programs [8]. The federal government also provides direct care to certain groups, including veterans and First Nation's peoples, through the First Nationals and Inuit Health Branch (FNIHB). Another role of the federal government is to ensure disease protection and to promote health issues [5].

The federal government demonstrates its financial commitment to Canada's human resources in health care by pledging transfer funds to the provinces and direct funding for various areas. For example, in the 2003 Health Care Renewal Accord, the federal government provided provinces and territories with a three-year CAD 1.5 billion Diagnostic/Medical Equipment Fund. This was used to support specialized staff training and equipment that improved access to publicly funded services [6].

The third group – private physicians – is generally not employed by the government, but rather is self-employed and works in a private practice. They deliver publicly-funded care to Canadian citizens. Physicians will negotiate fee schedules for their services with their provincial governments and then submit their claims to the provincial health insurance plan in order to receive their reimbursement [5].

The roles of nurses consist of providing care to individuals, groups, families, communities and populations in a variety of settings. Their roles require strong, consistent and knowledgeable leaders, who inspire others and support professional nursing practice. Leadership is an essential element for high-quality professional practice environments in which nurses can provide high-quality nursing care [9].

In most Canadian health care organizations, nurses manage both patient care and patient care units within the organization. Nurses have long been recognized as the mediators between the patient and the health care organization [10]. In care situations, they generally perform a coordinating role for all services needed by patients. They must be able to manage and process nursing data, information and knowledge to support patient care delivery in diverse care-delivery settings [10]. Workplace factors most valued by nurses include autonomy and control over the work environment, ability to initiate and sustain a therapeutic relationship with patients and a collaborative relationship with physicians at the unit level [11].

In addition to doctors and nurses, there are many more professionals involved in the health care process. Allied health care professionals can consist of pharmacists, dietitians, social workers and case managers, just to name a few. While much of the focus is on doctors and nurses, there are numerous issues that affect other health care providers as well, including workplace issues, scopes of practice and the impact of changing ways of delivering services [12]. Furthermore, with health care becoming so technologically advanced, the health care system needs an increasing supply of highly specialized and skilled technicians [12]. Thus we can see the various roles played by these five groups and how they work together to form the Canadian health care system.

Canada differs from other nations such as the United States of America for numerous reasons, one of the most important being the CHA. As previously mentioned, the CHA sets national standards for health care in Canada. The CHA ensures that all Canadian citizens, regardless of their ability to pay, will have access to health care services in Canada. "The aim of the CHA is to ensure that all eligible residents of Canada have reasonable access to insured health services on a prepaid basis, without direct charges at the point of service" [6].

Two of the most significant stipulations of the CHA read: "reasonable access to medically necessary hospital and physician services by insured persons must be unimpeded by financial or other barriers" and "health services may not be withheld on the basis of income, age, health status, or gender" [5]. These two statements identify the notable differences between the Canadian and American health care systems. That is, coverage for the Canadian population is much more extensive.

Furthermore in Canada, there has been a push towards a more collaborative, interdisciplinary team approach to delivering health care; this raises many new issues, one of which will involve successful knowledge transfer within these teams [13]. Effective knowledge management, which includes knowledge transfer, is increasingly being recognized as a crucial aspect of an organization's basis for long-term, sustainable, competitive advantage [34]. Even though health care in Canada is largely not for profit, there will still be the need for effective knowledge management practices to be developed and instituted. The introduction of interdisciplinary health teams in Canadian hospitals is a relatively new phenomenon and their connection to the knowledge management policies and agendas of governments and hospital administrations raises important questions about how such teams will work and to what extent they can succeed in dealing with the more difficult aspects of knowledge management, such as the transfer of tacit knowledge.

The multidisciplinary approach tends to be focused around specific professional disciplines, with health care planning being mainly top-down and dominated by medical professionals. Typically there is a lead professional (usually a physician) who determines the care and, if necessary, directs the patient to other health care specialists and allied professionals (aides, support workers). There is generally little involvement by the patient in the direction and nature of the care. Interdisciplinary health care is a patient-centred approach in which all those involved, including the patient, have input into the decisions being made.

The literature on teamwork and research on the practices in hospitals relating to multidisciplinary teams suggests that interdisciplinary teams face enormous challenges [13], therefore multidisciplinary teamwork will continue to be a vital part of the health care system. However, the goal of this teamwork should not be to displace one health care provider with another, but rather to look at the unique skills each one brings to the team and to coordinate the deployment of these skills. Clients need to see the health worker most appropriate to deal with their problem [14].

Some of the issues regarding the Canadian public system of health have been identified in the Mazankowski Report, which was initiated by Alberta's Premier Ralph Klein in 2000. Many issues have arisen since this time and have been debated among Canadians. One of the most contentious, for example, is the possibility of introducing a two-tier medical system. One tier of the proposed new system would be entirely government-funded through tax dollars and would serve the same purpose as the current publicly-funded system. The second tier would be a private system and funded by consumers [5].

However, the CHA and the Canadian Nurses Association (CNA) are critical of any reforms that pose a threat to the public health care system. It should be noted that although Canada purports to have a one-tier system, the close proximity of private, fee-for-service health care in the United States really creates a pay-as-you-go second tier for wealthy Canadians. In addition, many health care services such as most prescriptions and dental work are largely funded by individuals and/or private or employer paid insurance plans.

It is important to realize the differences between the proposed two-tier system and the current health care system. Presently, the public health care system covers all medically necessary procedures and the private sector provides 30% for areas such as dental care. With the new system, both public and private care would offer all services and Canadians would have the option of choosing between the two.

The proposal of the two-tier system is important because it highlights several important issues that concern many Canadians, mainly access to the system and cost reduction. Many Canadians believe the current public system is not sustainable and that a two-tiered system would force the public system to become more efficient and effective, given the competition of the private sector. However, the two-tiered system is not within the realm of consideration, since the majority of Canadians are opposed to the idea of a privatized system [5]. No proposals have come forward that show how a privately funded system would provide an equal quality of services for the same cost as the current publicly funded system.

#### United States of America

The health care system in the United States is currently plagued by three major challenges. These include: rapidly escalating health care costs, a large and growing number of Americans without health coverage and an epidemic of substandard care [15].

**Figure 3:**
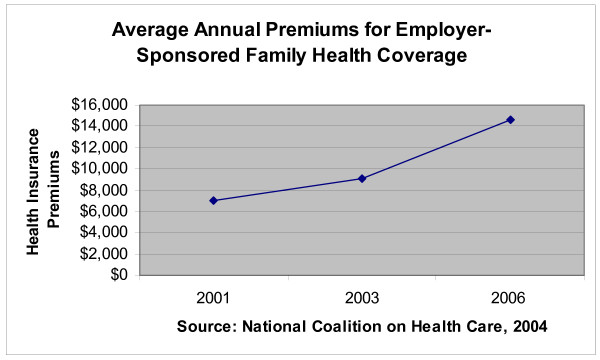
**The trend of the cost of employer-sponsored family health care coverage in the United States**. Source: National Coalition on Health Care 2004 pg.9. . Figure 3 illustrates the increase in health insurance premiums since 2001. These increases are making it more difficult for businesses to continue to provide health coverage for their employees and retirees [15].

Health insurance premiums in the United States have been rising at accelerating rates. The premiums themselves, as well as the rate of increase in premiums, have increased every year since 1998; independent studies and surveys indicate that this trend is likely to continue over the next several years [15]. As a result of these increases, it is more difficult for businesses to provide health coverage to employees, with individuals and families finding it more difficult to pay their share of the cost of employer-sponsored coverage [15]. The rising trend in the cost of employer-sponsored family health coverage is illustrated in Figure 3.

To help resolve this problem, health maintenance organizations (HMO) have been introduced, with the goal of focusing on keeping people well and out of hospitals in the hope of decreasing employer costs. HMOs are popular alternatives to traditional health care plans offered by insurance companies because they can cover a wide variety of services, usually at a significantly lower cost [16]. HMOs use "networks" of selected doctors, hospitals, clinics and other health care providers that together provide comprehensive health services to the HMOs members [16]. The overall trade-off with an HMO is reduced choice in exchange for increased affordability.

Another problem to address regarding the American health care system is the considerable and increasing number of Americans without health coverage. Health care coverage programs such as Medicare offer a fee-for-service plan that covers many health care services and certain drugs. It also provides access to any doctor or hospital that accepts Medicare [17]. Patients with limited income and resources may qualify for Medicaid, which provide extra help paying for prescription drug costs [17]. However, according to figures from the United States Census Bureau, the number of Americans without health coverage grew to 43.6 million in 2002; it is predicted that the number of uninsured Americans will increase to between 51.2 and 53.7 million in 2006 [15].

Those Americans without health care insurance receive less care, receive care later and are, on average, less healthy and less able to function in their daily lives than those who have health care insurance. Additionally, the risk of mortality is 25% higher for the uninsured than for the insured [15].

Despite excellent care in some areas, the American health care system is experiencing an epidemic of substandard care; the system is not consistently providing high-quality care to its patients [15]. There appears to be a large discrepancy between the care patients should be receiving and the care they are actually getting. The Institute of Medicine has estimated that between 44 000 and 98 000 Americans die each year from preventable medical errors in hospitals [15].

It is also useful to examine the demographic characteristics of those Americans more likely to receive substandard care. Research shows that those Americans with little education and low income receive a lower standard of care [18]. This finding may be explained by the fact that patients who have lower education levels tend to have more difficulty explaining their concerns to physicians, as well as eliciting a response for those concerns because health professionals often do not value their opinions [18].

### Case studies

As shown by the extensive literature, statistics and public opinion, there is a growing need for health care reform in the United States of America. There is a duty and responsibility of human resources professionals to attempt to elicit change and implement policies that will improve the health care system.

#### Case 1

It is informative to examine case studies in which human resources professionals have enacted positive change in a health care setting. One such case from 1995 is that of a mid-sized, private hospital in the New York metropolitan area. This case presents a model of how human resources can be an agent for change and can partner with management to build an adaptive culture to maintain strong organizational growth [19].

One of the initiatives made by human resources professionals in an attempt to improve the overall standard of care in the hospital was to examine and shape the organization's corporate culture. Steps were taken to define the values, behaviors and competences that characterized the current culture, and analyze these against the desired culture [19]. A climate survey was conducted in the organization; it became the goal of the human resources professionals to empower employees to be more creative and innovative [19]. To achieve this, a new model of care was designed that emphasized a decentralized nursing staff and a team-based approach to patient care. Nursing stations were redesigned to make them more accessible and approachable [19].

Human resources management also played an important role in investing in employee development. This was achieved by assisting employees to prepare and market themselves for internal positions and if desired, helping them pursue employment opportunities outside the organization [19]. This case makes obvious the important roles that human resources management can play in orchestrating organizational change.

#### Case 2

Another case study that illustrates the importance of human resources management to the health care system is that of The University of Nebraska Medical Center in 1995. During this period, the hospital administrative staff recognized a variety of new challenges that were necessitating organizational change. Some of these challenges included intense price competition and payment reform in health care, reduced state and federal funding for education and research, and changing workforce and population demographics [20]. The organizational administrators recognized that a cultural reformation was needed to meet these new challenges. A repositioning process was enacted, resulting in a human resources strategy that supported the organization's continued success [20]. This strategy consisted of five major objectives, each with a vision statement and series of action steps.

• Staffing: Here, the vision was to integrate a series of organization-wide staffing strategies that would anticipate and meet changing workforce requirements pertaining to staff, faculty and students. To achieve this vision, corporate profiles were developed for each position to articulate the core competences and skills required [20].

• Performance management: The vision was to hold all faculty and staff accountable and to reward individual and team performance. With this strategy, managers would be able to provide feedback and coaching to employees in a more effective and timely manner [20].

• Development and learning: The vision was to have all individuals actively engaged in the learning process and responsible for their own development. Various unit-based training functions were merged into a single unit, which defined critical technical and behavioral competencies [20].

• Valuing people: The vision was to have the hospital considered as a favored employer and to be able to attract and retain the best talent. To facilitate this vision, employee services such as child care and wellness were expanded [20].

• Organizational effectiveness. The vision was to create an organization that is flexible, innovative and responsive [20]. The developments of these human resources strategies were essential to the effectiveness of the organization and to demonstrate the importance of human resources in the health care industry.

Both these case studies illustrate that effective human resources management is crucial to health care in a practical setting and that additional human resources initiatives are required if solutions are to be found for the major problems in the United States health care system.

#### Germany

Approximately 92% of Germany's population receives health care through the country's statutory health care insurance program, Gesetzliche Krankenversicherung (GKV). GKV designed an organizational framework for health care in Germany and has identified and constructed the roles of payers, providers and hospitals. Private, for-profit companies cover slightly less than 8% of the population. This group would include, for example, civil servants and the self-employed. It is estimated that approximately 0.2% of the population does not have health care insurance [21]. This small fragment may be divided into two categories: either the very rich, who do not require it, or the very poor, who obtain their coverage through social insurance. All Germans, regardless of their coverage, use the same health care facilities. With these policies nearly all citizens are guaranteed access to high-quality medical care [22].

While the federal government plays a major part in setting the standards for national health care policies, the system is actually run by national and regional autonomous organizations. Rather than being financed solely through taxes, the system is covered mostly by health care premiums [22]. In 2003, about 11.1% of Germany's gross domestic product (GDP) went into the health care system [23] versus the United States, with 15% [24] and Canada at 9.9% [25]. However, Germany still put about one third of its social budget towards health care [22].

The supply of physicians in Germany is high, especially compared to the United States, and this is attributed largely to the education system. If one meets the academic requirements in Germany, the possibility to study medicine is legally guaranteed [26]. This has led to a surplus of physicians and unemployment for physicians has become a serious problem. In 2001, the unemployment rate for German physicians of 2.1% led many German doctors to leave for countries such as Norway, Sweden and the United Kingdom, all of which actively recruit from Germany [27].

Germany's strong and inexpensive academic system has led the country to educate far more physicians than the United States and Canada. In 2003, Germany had 3.4 practicing physicians per 1000 inhabitants [23], versus the United States, which had 2.3 practicing physicians per 1000 inhabitants in 2002 [24] and Canada, which had 2.1 practicing physicians per 1000 inhabitants in 2003 [25]. It is also remarkable that health spending per capita in Germany (USD 2996) [23] amounted to about half of health spending per capita in the United States (USD 5635) [24], and slightly less than Canada's health spending (USD 3003) [25]. This clearly demonstrates the Germans' strength regarding cost containment.

There are several issues that physicians face in the German health care system. In a 1999 poll, 49.9% of respondents said they were very or fairly satisfied with their health care system, while 47.7% replied they were very or fairly dissatisfied with it [28]. Furthermore, the degree of competition between physicians is very high in Germany and this could lead to a reduction in physician earnings. Due to this competition, many younger physicians currently face unemployment. The German law also limits the number of specialists in certain geographical areas where there are issues of overrepresentation [22]. Thus, the oversupply of physicians in Germany leads to many challenges, including human resources management in the health care system.

In Germany a distinction is made between office-based physicians and hospital-based physicians. The income of office-based physicians is based on the number and types of services they provide, while hospital-based physicians are compensated on a salary basis. This division has created a separated workforce that German legislation is now working to eliminate by encouraging the two parties to work together, with the aim of reducing overall medical costs [22].

### Developing countries

Accessing good-quality health care services can be incredibly arduous for those living in developing countries, and more specifically, for those residing in rural areas. For many reasons, medical personnel and resources may not be available or accessible for such residents. As well, the issue of migrant health care workers is critical. Migrant health workers can be defined as professionals who have a desire and the ability to leave the country in which they were educated and migrate to another country. The workers are generally enticed to leave their birth country by generous incentive offers from the recruiting countries [29].

Developing countries struggle to find means to improve living conditions for their residents; countries such as Ghana, Kenya, South Africa and Zimbabwe are seeking human resources solutions to address their lack of medically trained professionals. Shortages in these countries are prevalent due to the migration of their highly educated and medically trained personnel.

Professionals tend to migrate to areas where they believe their work will be more thoroughly rewarded. The *International Journal for Equity in Health (2003) *suggested that those who work in the health care profession tend to migrate to areas that are more densely populated and where their services may be better compensated. Health care professionals look to areas that will provide their families with an abundance of amenities, including schools for their children, safe neighborhoods and relatives in close proximity. For medical professionals, the appeal of promotions also serves as an incentive for educating oneself further [30]. As one becomes more educated, the ability and opportunity to migrate increases and this can lead to a further exodus of needed health care professionals.

These compelling reasons tend to cause medical professionals to leave their less-affluent and less-developed areas and migrate to areas that can provide them with better opportunities. This has caused a surplus in some areas and a huge deficit in others. This epidemic can be seen in nations such as Nicaragua. Its capital city, Managua, holds only one fifth of the country's population, yet it employs almost 50% of the medically trained health care workers. The same situation can be found in other countries, such as Bangladesh, where almost one third of the available health personnel are employed "in four metropolitan districts where less than 15% of the population lives" [30]. Clearly this presents a problem for those living outside these metropolitan districts.

**Figure 4:**
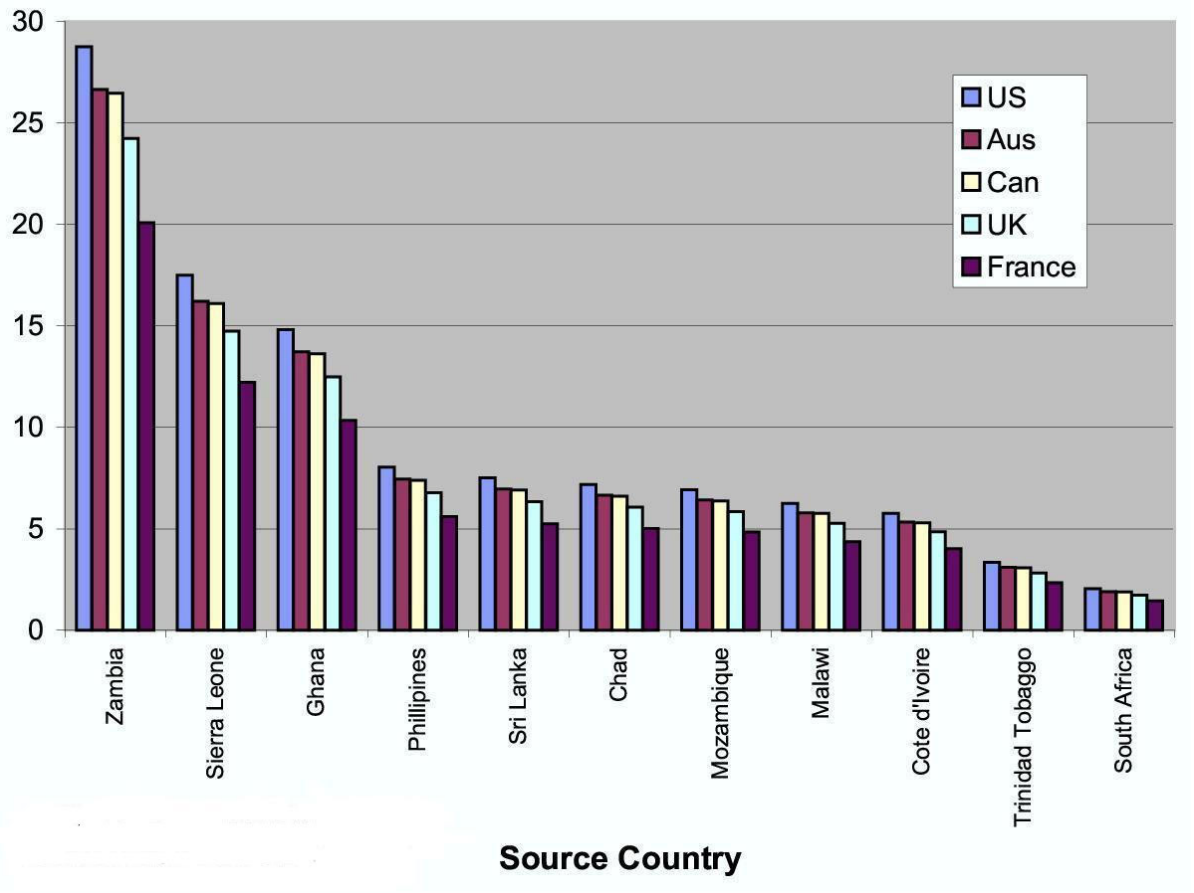
**Ratio of nurse wages (PPP USD), destination country to source country**. Source: Vujicic M, Zurn P, Diallo K, Orvill A, Dal Poz MR 2004. . Figure 4 shows the difference between the wage in the source country and destination country for nurses. This difference is also known as the "wage premium" [29].

**Figure 5:**
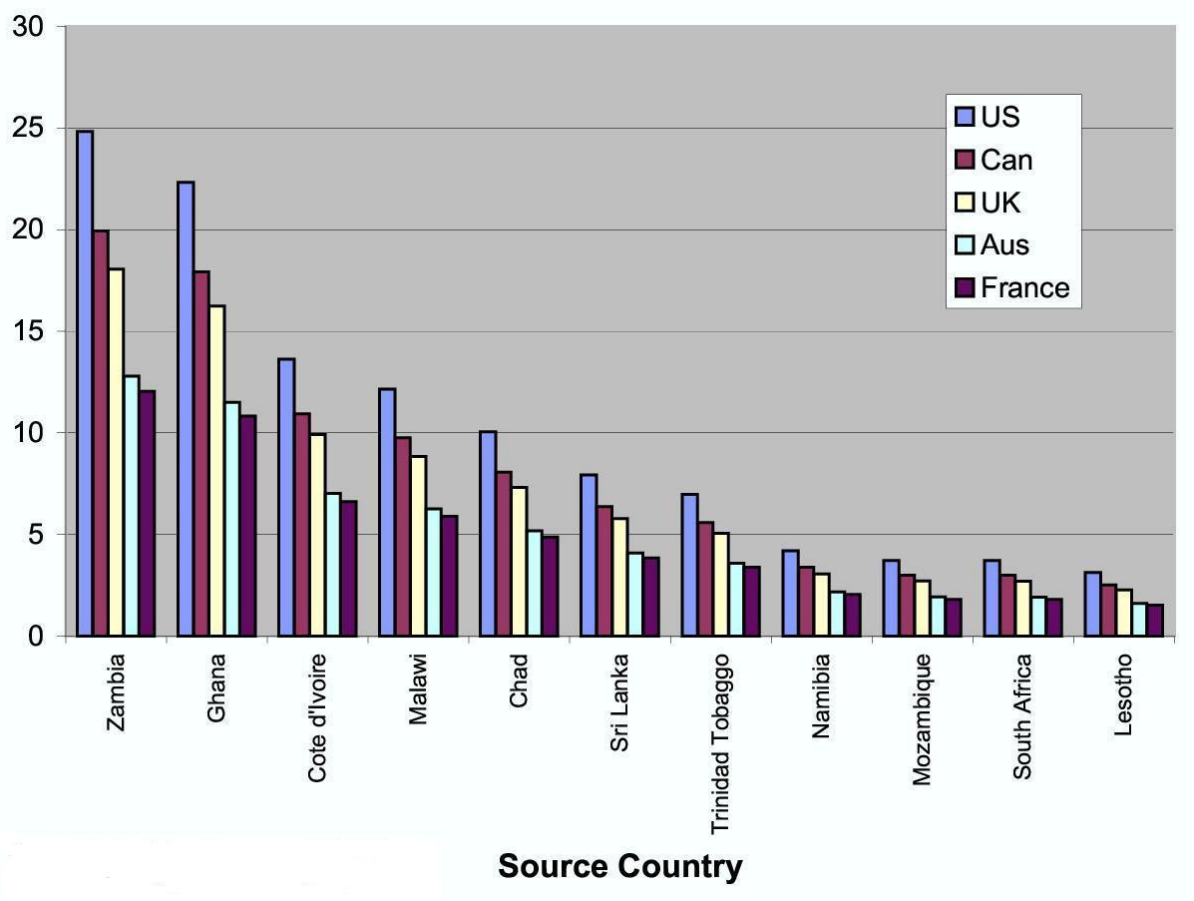
**Ratio of physician wages (PPP USD), destination country to source country**. Source: Vujicic M, Zurn P, Diallo K, Orvill A, Dal Poz MR 2004. . Figure 5 shows the difference between the wage in the source country and destination country for physicians [29].

Other possible explanations put forth by Dussault and Franceschini, both of the Human Development Division of the World Bank Institute, include "management style, incentive and career structures, salary scales, recruitment, posting and retention practices" [31]. Salary scales can differ quite drastically between originating and destination countries, which are shown in Figures 4 and 5. They also state that in developing countries the earning potential one would see in more affluent or populated urban areas is much higher than one would expect to earn in rural areas.

As more health professionals emigrate to urban areas, the workloads for those in the rural areas greatly increase. This leads to a domino effect, in that those in such dire situations look for areas where they may be able to find more satisfactory and less demanding working conditions [31]. Vujicic et al. (2004) summarizes numerous variables that influence the migration pattern and has created a formula to express their impact. It is possible to quantify the factors, and human resources professionals need to look at the costs and benefits of altering the factors so that the migration pattern is more favorable. This formula is expressed as the results shown in Table 1, which shows the different reasons for one to migrate in terms of the popularity of a given reason.

**Table 1:** Factors influencing health care professionals' intent to migrate, reason for migrating and willingness to remain in their home country. Source: Vujicic M, Zurn P, Diallo K, Orvill A, Dal Poz MR 2004 .

	**For what reasons do you intend to leave your home country?**	**For what reasons did you leave your home country?**	**What would make you remain in your home country?**
**Cameroon**	Upgrade qualifications (85%) Gain experience (80%) Lack of promotion (80%) Living conditions (80%)	Recruited (29%) Gain experience (28%) Better pay (27%) Living conditions (19%)	Salary (68%) Continuing education (67%) Working environment (64%) Health care system management (55%)
**Ghana**		Gain experience (86%) Lack of promotion (86%) Despondency (86%) Living conditions, Economic decline (71%)	Salary (81%) Work environment (64%) Fringe benefits (77%) Resources in health sector (70%)
**Senegal**	Salary (89%) N/a N/a N/a		Work environment (n/a) Salary (n/a) Better career path (n/a) Benefits (n/a)
**South Africa**	Gain experience (43%) Violence and crime (38%) Heavy workload (41%) Declining health service (38%)		Salary (78%) Work environment (68%) Fringe benefits (66%) Workload (59%)
**Uganda**	Salary (72%) Living conditions (41%) Upgrade qualifications (38%) Gain experience (24%)	Salary (55%) Economic decline (55%) Save money (54%) Declining health service (53%)	Salary (84%) Fringe benefits (54%) Work environment (36%) Workload (30%)
**Zimbabwe**	All factors	All factors	All factors

There is a tendency for developed countries faced with decreasing numbers of nationally trained medical personnel to recruit already-trained individuals from other nations by enticing them with incentives. Zimbabwe has been particularly affected by this problem. In 2001, out of approximately 730 nursing graduates, more than one third (237) of them relocated to the United Kingdom [29]. This was a dramatic increase from 1997, when only 26 (approximately 6.2%) of the 422 nursing program graduates migrated to the United Kingdom [29]. This leads to the loss of skilled workers in developing countries and can be very damaging, since the education systems in developing countries are training individuals for occupations in the medical profession, yet are not able to retain them [29].

Countries that have the capacity to educate more people than necessary in order to meet their domestic demand have tried to counterbalance this problem by increasing their training quota. Vujicic et al. (2004) identify that "the Philippines has for many years trained more nurses than are required to replenish the domestic stock, in an effort to encourage migration and increase the level of remittance flowing back into the country" [29].

Developed countries attract internationally trained medical professionals for many reasons. To begin with, "political factors, concerns for security, domestic birth rates, the state of the economy and war (both at home and abroad)" [26] influence the number of people that will be allowed or recruited into a country. Also, due to the conditions of the labor market compared to the demand in developed countries, governments may make allowances to their strict policies regarding the type of and number of professionals they will allow into their country [29]. This can be seen in a Canadian example:

Canada maintains] a list of occupations within which employment vacancies [are] evident. Potential immigrants working in one of these [listed] occupations would have a much higher chance of being granted entry than if they worked in a non-listed occupation [29].

Though Canada attracts internationally trained medical professionals, those employment vacancies may not always be open. Although there may be up to 10 000 international medical graduates (IMG) in Canada, many are not legally allowed to practice. Many immigrants cannot afford the costs of retraining and may be forced to find a new job in a completely unrelated field, leaving their skills to go to waste [32]. In 2004, Ontario had between 2000 and 4000 IMGs looking for work in medical fields related to their training and background [33]. That year, IMG Ontario accepted 165 IMGs into assessment and training positions, which was a 50% increase over the last year, and a 600% increase from the 24 positions in 1999 [33].

Another appeal for developed countries with regard to foreign trained health care professionals is that they may be less of a financial burden to the host country than those trained domestically. This is because educational costs and the resources necessary for training are already taken care of by the international medical schools and governments [29]. Though these reasons may make recruiting foreign medical professionals seem appealing, there are still ongoing debates as to whether those trained outside the host country are equally qualified and culturally sensitive to the country to which they migrate. Developing countries are addressing these concerns by establishing health professional training programs similar to those in developed countries [29]. These practices can be seen in, "the majority of nursing programs in Bangladesh, the Philippines and South Africa [which] are based on curricula from United Kingdom or USA nursing schools" [29]. Because of these actions, those who are trained may be more likely to leave and use their skills where they will be recognized and more highly rewarded.

There are also ethical considerations when examining the practice of recruiting health care professionals, particularly if they are recruited from regions or countries where health care shortages already exist. The rights of individuals to move as they see fit may need to be balanced against the idea of the greater good of those left behind.

Due to the shortages, it has been found the level of health service in rural or poor areas has decreased, leading to lower quality and productivity of health services, closure of hospital wards, increased waiting times, reduced numbers of available beds for inpatients, diversion of emergency department patients and underuse of remaining personnel or substitution with persons lacking the required skills for performing critical interventions [30].

The article "Not enough here, too many there: understanding geographical imbalances in the distribution of the health workforce" (2003), states that a reduced number of health care workers in a given area has a direct effect on the life expectancy of its residents. For example, in the rural areas of Mexico, life expectancy is 55 years, compared to 71 years in the urban areas. Additionally, in "the wealthier, northern part of the country, infant mortality is 20/1000 as compared to more than 50/1000 in the poorer southern states" [31].

### Globalization – a common thread

While the issues raised in this article are common to many countries, the approaches taken to address them may not be the same in each country. Factors affecting the approaches that can be taken, some of which have been raised, include demographics, resources and philosophical and political perspectives. However, an overarching issue that affects not only health care but many other areas is that of globalization itself.

Different countries have traditionally had different perspectives on health care that have influenced their approaches to health care delivery. In Canada for example, health care is considered a right; its delivery is defined by the five main principles of the Canada Health Act, which officially precludes a significant role for private delivery of essential services. In the United States, health care is treated more as another service that, while it should be accessible, is not considered a right. Therefore there is a much larger private presence in health care delivery the United States than there is in Canada. In other parts of the world, the approach to health care falls between these perspectives.

As the move towards globalization for many goods and services increases, countries will have to consider how this will affect their approaches to health care delivery. As mentioned earlier, there is already a degree of labor mobility within a country that affects the quality and availability of health care services. There is also already a degree of international mobility of health care workers, as shown by the number of workers recruited developed countries.

While the international mobility of labor is generally not as unencumbered as that for goods and capital, that may be changing as more and more regional free trade agreements are considered. Canada, the United States and Mexico have NAFTA (North American Free Trade Agreement), Europe has the EU (European Union) and talks are under way to consider expanding the NAFTA agreement to include Central and South America, to expand EU membership and to consider an Asian trading bloc including China and India.

If health care becomes a part of these new trade agreements, countries will be obliged to treat health care delivery according to the rules of the agreement. Using the NAFTA as an example, if health care is included, governments could not treat domestic providers more favorably than foreign firms wanting to deliver services. In Canada the concern is that it would mean the end of the Canada Health Act, since NAFTA would allow private, for-profit American or Mexican firms to open.

All five issues raised in this research would be affected by the increase in international trade agreements that included health care. Therefore, governments, health care providers and human resources professionals cannot ignore this important consideration and trend when examining solutions to the issues. Depending upon their relative negotiation strengths and positions, some countries may not benefit as much as others with these agreements.

For example, it is more likely that countries with well-developed private, for-profit, health care expertise, such as the United States, would expand into developing countries rather than the other way around. If there is an increased ability for labor mobility, then it is likely that health care professionals in the poorer, developing countries would move to where the opportunities are better. We already see this internally in the move from rural to urban centers; this would likely continue if the health care professionals had the opportunity to move out of country to where they could have greater financial rewards for their expertise.

When considering the countries examined in this paper, it is likely that Canada and the United States would initially be the two most likely to move towards a more integrated approach to health care delivery. There is already a trade agreement in place, many of the factors influencing health care are similar (demographics, training, level of economic development, geography, cultural factors) and they are currently each other's largest trading partners. While the current agreement, which includes Mexico, does not cover health care, there is pressure to broaden the agreement to include areas not currently covered. If this happens, human resources professionals will have to increase their understanding of what the new health care delivery realities could be. For example, if the move is more towards the Canadian example of a largely not-for-profit, mainly publicly-funded health care delivery system, then it will be more of an adjustment for the American professionals.

However, the likelihood of the Canadian approach to health care's being adopted in the United States is very slim. During the presidency of Bill Clinton, the government attempted to introduce a more universal health care delivery system, which failed completely. Even though there are over 40 million Americans with no health care coverage, the idea of a universal, publicly-funded system went nowhere. Also, within Canada there is increasing pressure to consider a more active role for private health care delivery. Therefore, it is more likely that Canadian health care and human resource professionals will have to adapt to a style more like the American, privately delivered, for-profit approach.

If this is the direction of change, human resources professionals in Canada will need to adjust how they approach the challenges and new realities. For instance, there would likely be an increased role for insurance companies and health maintenance organizations (HMO) as they move towards the managed care model of the United States. With an HMO approach, financial as well as health needs of the patients are considered when making medical decisions. An insured patient would select from the range of services and providers that his/her policy covers and approves. Human resources professionals would need to work with a new level of administration, the HMO, which currently does not exist to any significant degree in Canada.

As mentioned earlier, it is likely that developing countries would be receiving health care models and approaches from developed countries rather than the other way around. In particular, a country such as the United States that has a strong, private, for-profit approach already in place would likely be the source from which the health care models would be drawn. Therefore, health care, as well as human resources professionals in those countries, would also need to adapt to these new realities.

In Germany, where there is currently an oversupply of physicians, a move towards a more global approach to health care delivery, through increased trade agreements, could result in even more German health care professionals' leaving the country. The challenge to be addressed by human resource professionals within the German health care system in this situation would be to prevent, or slow, the loss of the best professionals to other countries. Spending public resources in educating professionals only to have significant numbers of them leave the country is not a financially desirable or sustainable situation for a country.

## Discussion

While examining health care systems in various countries, we have found significant differences pertaining to human resources management and health care practices. It is evident that in Canada, CHA legislation influences human resources management within the health care sector. Furthermore, the result of the debate on Canada's one-tier versus two-tier system may have drastic impacts on the management of human resources in health care. Additionally, due to a lack of Canadian trained health professionals, we have found that Canada and the United States have a tendency to recruit from developing countries such as South Africa and Ghana, in order to meet demand.

Examination of the relationship between health care in the United States and human resources management reveals three major problems: rapidly escalating health care costs, a growing number of Americans without health care coverage and an epidemic regarding the standard of care. These problems each have significant consequences for the well-being of individual Americans and will have devastating affects on the physical and psychological health and well-being of the nation as a whole.

The physical health of many Americans is compromised because these factors make it difficult for individuals to receive proper consultation and treatment from physicians. This can have detrimental effects on the mental state of the patient and can lead to large amounts of undue stress, which may further aggravate the physical situation.

Examining case studies makes it evident that human resources management can and does play an essential role in the health care system. The practices, policies and philosophies of human resources professionals are imperative in developing and improving American health care. The implication is that further research and studies must be conducted in order to determine additional resource practices that can be beneficial to all organizations and patients.

Compared to the United States, Canada and developing countries, Germany is in a special situation, given its surplus of trained physicians. Due to this surplus, the nation has found itself with a high unemployment rate in the physician population group. This is a human resources issue that can be resolved through legislation. Through imposing greater restrictive admissions criteria for medical schools in Germany, they can reduce the number of physicians trained. Accompanying the surplus problem is the legislative restriction limiting the number of specialists allowed to practice in geographical areas. These are two issues that are pushing German-trained physicians out of the country and thus not allowing the country to take full advantage of its national investment in training these professionals.

Developing countries also face the problem of investing in the training of health care professionals, thus using precious national resources, but losing many of their trained professionals to other areas of the world that are able to provide them with more opportunities and benefits. Human resources professionals face the task of attempting to find and/or retain workers in areas that are most severely affected by the loss of valuable workers.

Human resources management plays a significant role in the distribution of health care workers. With those in more developed countries offering amenities otherwise unavailable, chances are that professionals will be more enticed to relocate, thus increasing shortages in all areas of health care. Due to an increase in globalization, resources are now being shared more than ever, though not always distributed equally.

### Human resources implications of the factors

While collectively the five main areas addressed in the article represent health care issues affecting and affected by human resources practices, they are not all equal in terms of their influence in each country. For instance, in Canada there are fewer health care issues surrounding the level of economic development or migration of health workers, whereas these issues are much more significant in developing countries. In the United States, the level of economic development is not a significant issue, but the accessibility of health care based upon an individual's financial situation certainly is, as evidenced by the more than 40 million Americans who have no health care coverage. Germany's issues with the size of its health care worker base have to do with too many physicians, whereas in Canada one of the issues is having too few physicians. Table 2 summarizes some of the implications for health care professionals with regard to the five main issues raised in the article. One of the main implications of this paper, as shown in Table 2, is that HRP will have a vital role in addressing all the factors identified. Solutions to health care issues are not just medical in nature.

**Table 2:** Human resources implications of the factors

	**Countries**			
**Factors**	**Canada**	**United States**	**Germany**	**Developing countries**

*Number, composition and distribution of health care workers*	Human resources professionals (HRP) will need to assess needs throughout all regions of Canada. Given that health care delivery is a provincial and territorial responsibility, HRP will need to work with and through the 13 different provinces and territories in Canada. This will require a greater understanding of regional issues, practices, etc.	While not formally entrenched in legislature as it is in Canada, health delivery is a more regional than national and therefore HRP will have many of the same issues as they have in Canada in terms of working at the state and local levels.	As cited in the research, Germany currently has an oversupply of physicians and health other health professionals in certain regions. This imbalance will require HRP to work with the regional authorities to better understand the needs of specific regions and help plan for a better match between supply of and demand for health professionals.	Developing countries face significant challenges in all five areas discussed in this paper. Therefore, as described in reference to Canada, the United States and Germany, HRP will have an active role in all areas as well. It is not just a matter of more health resources for developing countries, since these resources must be managed efficiently and effectively. Since one of the largest and most complex health care input are the human resources, it is clear that as developing countries increase the number of workers, HRP will have an active and important role to play.
*Workforce training issues*	As shown in the research, a move towards a more interdisciplinary approach to health care delivery will require new skills on the part of health workers. HRP will have a significant role in helping to create a culture that encourages this type of health care delivery.	The case studies cited in this paper show that HRP will have a significant role in helping to develop the appropriate culture in health organizations to ensure that delivery is as effective and efficient as possible.	With the current oversupply of some health workers, HRP have the opportunity to develop and provide input into planning for future training that more accurately reflects current and anticipated needs. As in Canada and the United States, the move towards a more interdisciplinary approach will also be reflected here since the complexity of health care means that no one person can have all of the answers. Therefore, HRP will need to provide input on how to make this new approach work.	With more limited resources available, workforce training issues can become challenging and HRP will have to help develop strategies that are appropriate and sustainable. These approaches could include an increased use of technology or a broadened role for different health workers such as nurses. HRP will have to work with existing health workers to help integrate these and other new approaches to how workers are trained.
*Migration of health workers*	As with many countries, there are challenges in meeting the health needs of the remote areas of Canada. HRP will need to work with the provinces to develop programmes and incentives to encourage health workers to consider moving to these areas.	The United States faces similar situations to that of Canada in this regard and HRP will have to work with the state officials to develop programmes and incentives to encourage health workers to consider moving to these areas.	By addressing and helping to better balance the supply of health workers with the demand, there will be less migration of workers out of Germany. There are significant costs to a country in training a health care professional and if workers leave after being trained, the country will not receive any benefit from its training investment. HRP can help to ensure that there is less migration of workers out of Germany by working at the strategic planning levels to help better match supply and demand.	This is an especially challenging area in developing countries, not only because of the extreme differences between the rural and urban areas, but also because of the increasing pressure from other countries to "poach" health care workers. By helping to develop policies and strategies such as those described above in "Workforce Training Issues", HRP can help reduce the migration of health workers from where they are needed.
*Level of economic development in a country*	Although Canada is an economically well-developed country, it, too, is facing financial pressures in the area of health spending. As discussed earlier in this paper, HRP will need to be involved at the strategic level of health planning in order to be able to influence discussions on spending priorities in this area. HRP should no longer be seen as just implementers of policies developed by others.	The United States, one of the wealthiest countries in the world, does not lack the means to obtain the latest health resources. Rather, the issue is that individuals do not always have the financial ability to gain access to these resources. This is shown by the fact that over 40 million Americans have no health care coverage. HRP will need to work with health care professionals at a senior, strategic level to address this pressing problem.	Germany is economically well-developed, but it, too, is facing economic realities and the pressures of increasing health care costs. HRP can contribute to the development of a more efficient and effective health care delivery system by being involved at the strategic level rather than just being implementers of policies developed by others.	This is a significant issue in developing countries where the resources for even the most basic health care needs may be difficult to obtain and sustain. By helping the health system to become more efficient and effective, HRP will help these countries make the most of the resources they have.
*Sociodemographic, geographical and cultural*	While there are not many significant geographical or cultural issues, Canada is facing an ageing population, which will make significant and increasing health care demands. HRP will need to play an active role at the strategic levels in order to ensure their skills, abilities and contributions are considered at this level.	The United States also faces an ageing population, which will make significant and increasing health care demands. As in Canada, HRP will need to play an active role at the strategic levels.	Germany also faces the challenges of an ageing population and therefore has to make the health care system as efficient and effective as possible. HRP have the opportunity and responsibility to play an active role at the strategic level.	Countries such as Canada and the United States are very similar in terms of these factors, so approaches that work in one country would not require much adjustment to work in another. Germany, while not being as similar as Canada and the United States are to each other, is a developed country and would be able to employ many of the human resource approaches to health care that would work in Canada and the United States. HRP have a great opportunity to identify and factor in the socio-demographic, geographical and cultural differences found in developing countries, since that is what they are trained to do. HRP will have a vital role in ensuring that approaches that may work in other countries are not applied without consideration of these differences.

### Policy approaches in a global approach to health care delivery

As mentioned at the start of this paper, there are three main health system inputs: human resources, physical capital and consumables. Given that with sufficient resources any country can obtain the same physical capital and consumables, it is clear that the main differentiating input is the human resources. This is the input that is the most difficult to develop, manage, motivate, maintain and retain, and this is why the role of the human resources professional is so critical.

The case studies described earlier showed how human resources initiatives aimed at improving organizational culture had a significant and positive effect on the efficiency and effectiveness of the hospitals studied. Ultimately all health care is delivered by people, so health care management can really be considered people management; this is where human resources professionals must make a positive contribution.

Human resource professionals understand the importance of developing a culture that can enable an organization to meet its challenges. They understand how communities of practice can form around common goals and interests, and the importance of aligning these to the goals and interests of the organization.

Given the significant changes that globalization of health care can introduce, it is important that human resources professionals be involved at the highest level of strategic planning, and not merely be positioned at the more functional, managerial levels. By being actively involved at the strategic levels, they can ensure that the HR issues are raised, considered and properly addressed.

Therefore, human resources professionals will also need to have an understanding not only of the HR area, but of all areas of an organization, including strategy, finance, operations, etc. This need will have an impact on the educational preparation as well as the possible need to have work experience in these other functional areas.

## Conclusion

We have found that the relationship between human resources management and health care is extremely complex, particularly when examined from a global perspective. Our research and analysis have indicated that several key questions must be addressed and that human resources management can and must play an essential role in health care sector reform.

The various functions of human resources management in health care systems of Canada, the United States of America, Germany and various developing countries have been briefly examined. The goals and motivations of the main stakeholders in the Canadian health care system, including provincial governments, the federal government, physicians, nurses and allied health care professionals, have been reviewed. The possibility of a major change in the structure of Canadian health care was also explored, specifically with regard to the creation of a two-tier system. The American health care system is currently challenged by several issues; various American case studies were examined that displayed the role of human resources management in a practical setting. In Germany, the health care situation also has issues due to a surplus of physicians; some of the human resources implications of this issue were addressed. In developing countries, the migration of health workers to more affluent regions and/or countries is a major problem, resulting in citizens in rural areas of developing countries experiencing difficulties receiving adequate medical care.

Since all health care is ultimately delivered by and to people, a strong understanding of the human resources management issues is required to ensure the success of any health care program. Further human resources initiatives are required in many health care systems, and more extensive research must be conducted to bring about new human resources policies and practices that will benefit individuals around the world.

## Competing interests

The author(s) declare that they have no competing interests.

## Authors' contributions

SK conceived the paper, worked on research design, did data analysis and led the writing of the paper. CO, JH, MS and RL all actively participated in data analysis, manuscript writing and review. All authors read and approved the final manuscript.
